# Combining antiangiogenic therapy with neoadjuvant chemotherapy increases treatment efficacy in stage IIIA (N2) non-small cell lung cancer without increasing adverse effects

**DOI:** 10.18632/oncotarget.11547

**Published:** 2016-08-23

**Authors:** Xiaoliang Zhao, Yanjun Su, Jian You, Liqun Gong, Zhenfa Zhang, Meng Wang, Zhenqing Zhao, Zhen Zhang, Xiaolin Li, Changli Wang

**Affiliations:** ^1^ Department of Lung Cancer, Tianjin Medical University Cancer Institute and Hospital, Tianjin 300060, P.R. China; ^2^ Tianjin Lung Cancer Center, Tianjin 300060, P.R China; ^3^ Tianjin Key Laboratory of Cancer Prevention and Therapy, Tianjin 300060, P.R China; ^4^ National Clinical Research Center for Cancer, Tianjin 300060, P.R China

**Keywords:** recombinant human endostatin, neoadjuvant chemotherapy, non-small cell lung cancer

## Abstract

To evaluate the safety and efficacy of combining Endostar antiangiogenic therapy with neoadjuvant chemotherapy for the treatment of stage IIIA (N2) NSCLC, we conducted a randomized, controlled, open-label clinical study of 30 NSCLC patients. Patients were randomly assigned to the test or control groups, which received either two cycles of an NP neoadjuvant chemotherapy regimen combined with Endostar or the NP regimen alone, respectively, at a 2:1 ratio. Efficacy was assessed after 3 weeks, and surgical resection occurred within 4 weeks, in the 26 patients who successfully completed treatment. While total response rates (RR) and clinical benefit rates (CBR) did not differ between the experimental groups, total tumor regression rates (TRR) were higher in the test group than in the control group. Median DFS and OS also did not differ between the test and control groups. Clinical perioperative indicators, including intraoperative blood loss, number of dissected lymph node groups, duration of postoperative indwelling catheter use, and time to postoperative discharge, were comparable in the test and control groups. Finally, hematological and non-hematological toxicities and postoperative pathological indicators, including down-staging ratio, complete resection ratio, and metastatic lymph node ratio, also did not differ between the groups. Overall, combining Endostar with NP neoadjuvant chemotherapy increased therapeutic efficacy without increasing adverse effects in stage IIIA-N2 NSCLC patients. This study is registered with ClinicalTrials.gov (number NCT02497118).

## INTRODUCTION

Lung cancer is a leading cause of death related to malignant tumors. Mediastinal lymph node-positive stage IIIA non-small cell lung cancer (NSCLC), confirmed either preoperatively or by postoperative pathology, is associated with poor prognosis [1]. Neoadjuvant chemotherapies modestly suppress stage IIIA NSCLC in some patients [2–4]. The antiangiogenic agent Endostar specifically inhibits the migration of, and induces apoptosis in, endothelial cells in new vessels [5–6]. Anti-neovascularization treatments may act synergistically with chemotherapy to improve the ability of treatments to reduce tumor burdens. Studies have found that, compared to chemotherapy alone, combining Endostar with an NP neoadjuvant chemotherapy regimen markedly improves response rates, clinical benefit rates, and time to progression in advanced NSCLC patients [7–8]. While it is possible that using Endostar concurrently with neoadjuvant therapy could improve response rates, clinical benefit rates, and surgical resection rates, few reports have examined the efficacy and surgical safety of this combined treatment. We therefore conducted a randomized, controlled, open-label clinical study at the Tianjin Medical University Cancer Institute and Hospital between April 2011 and December 2013 to evaluate the perioperative safety and efficacy of NP chemotherapy alone and in combination with Endostar for the treatment of stage IIIA(N2) NSCLC.

## RESULTS

### Efficacy of the combined endostar and NP regimen

**Figure 1:**
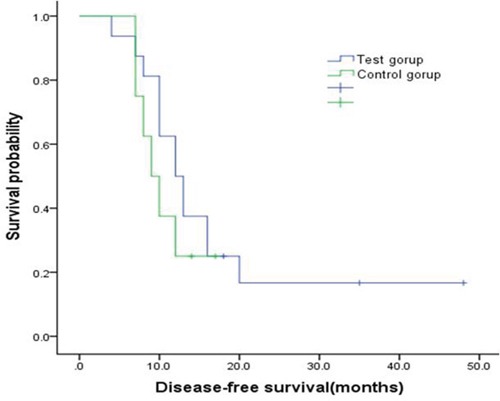
Kaplan-Meier curve for disease free survival by two group Compared with the control group, median DFS was extended by about 3 months in the test group (12 months vs 9 months), indicating superiority in survival which however presented no statistically significant differences (p=0.41).

**Figure 2:**
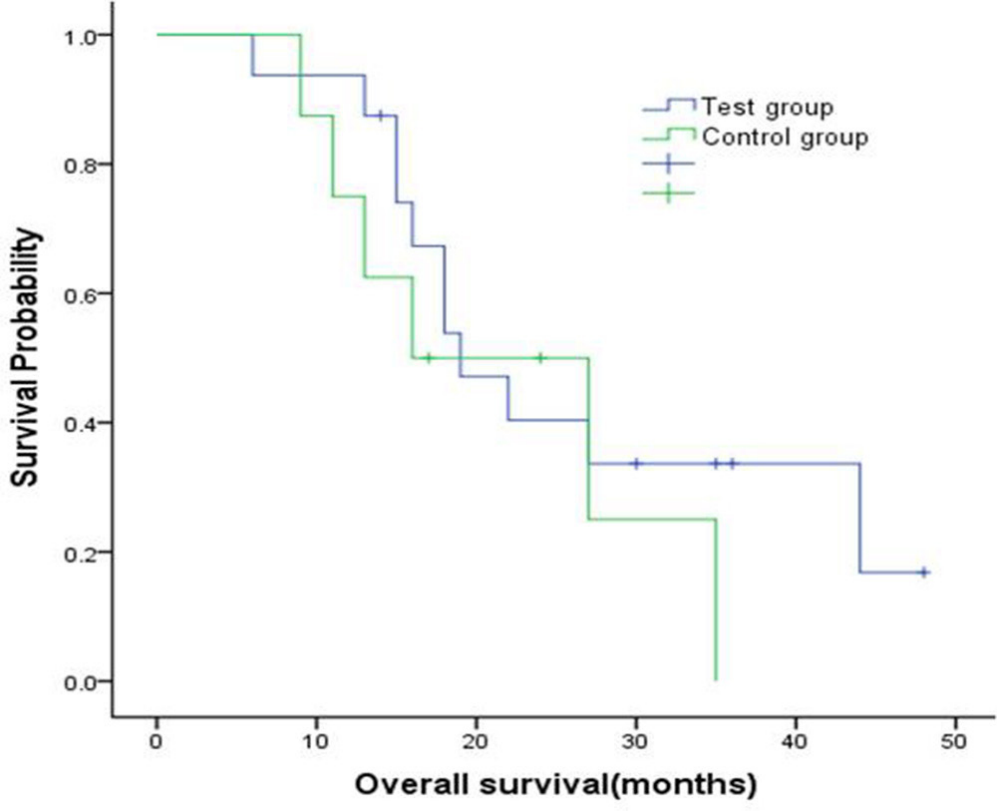
Kaplan-Meier curve for overall survival by two group Compared with the control group, median OS was extended by about 3 months in the test group (19 months vs 16 months), indicating superiority in overall survival which however presented no statistically significant differences (p=0.39).

Comparisons of treatment efficacy between the test (Endostar and NP) and control (NP alone) groups are shown in Table 1. We found trends towards increases in total response rates (RR) and clinical benefit rates (CBR) by about 10% and 27.5%, respectively (*p*>0.05), and tumor regression rates (TRR) increased by about 12% (*p*<0.05), in the test group compared to the control group. Compared to the control group, median disease-free survival (DFS) (12 months, 95%CI 9.06 to 14.94 vs 9 months, 95%CI 6.23 to 11.77) and median overall survival (OS) (19 months, 95%CI 13.37 to 24.63 vs 16 months, 95%CI 3.07 to 28.94) both tended to increase by approximately 3 months in the test group, although these differences were not statistically significant (Figures 1 and 2).

**Table 1:** Comparison of primary efficacy variables in the two groups

Group	N	Efficacy assessment				RR	CBR	mDFS	mOS	TRR
		CR	PR	SD	PD					
Test	16	0	8	6	2	50%	87.5%	12	19	19.7%
Control	10	0	4	2	4	40%	60%	9	16	7.1%
*p* value	-	1.0	1.0	0.68	0.37	1.0	0.76	0.41	0.39	0.036

### Safety and adverse reactions

The 26 patients for whom it was possible to evaluate adverse reactions received a total of 52 cycles of treatment without experiencing any serious adverse events or death. No other treatment-related adverse events, such as angina, arrhythmia, or ECG abnormalities, were observed in the test or control groups (Table 2).

**Table 2:** Comparison of adverse events in the two groups

Adverse event	Test group		Control group		*p* value
	Grade I-II	Grade I-IV	Grade I-II	Grade I-IV	
Leucopenia	6 (37.5%)	8 (50%)	2 (20%)	2 (20%)	0.68
Anemia	2 (12.5%)	2 (12.5%)	1 (10%)	1 (10%)	0.60
Thrombocytopenia	2 (12.5%)	2 (12.5%)	0 (0)	0 (0)	1.00
Nausea & vomiting	7 (43.8%)	7 (43.8%)	2 (20%)	2 (20%)	0.69
Elevated aminotransferase	3 (18.8%)	3 (18.8%)	2 (20%)	2 (20%)	1.00
Elevated total bilirubin	1 (6.75%)	1 (6.75%)	0 (0)	0 (0)	1.00

### Perioperative conditions

Efficacy was evaluated after patients received 2 treatment cycles; with the exception of two control group patients with in which tumors progressed to stage IV and were surgically unresectable, the remaining 24 patients underwent standard radical lung cancer resection (lung lobectomy and regional lymph node dissection). There were no significant differences in intraoperative blood loss, number or groups of lymph dissected nodes, duration of postoperative indwelling catheter use, mean daily chest drainage volume, or mean duration of postoperative hospitalization between the control and test groups (Table 3).

**Table 3:** Perioperative surgical conditions

	Test group (n=16)	Control group (n=8)	*p* value
Intraoperative blood loss (mL)	195±34	226±22	0.11
Lymph nodes dissected	21.4±9.92	25.0±11.0	0.28
Lymph node groups dissected	8.56±3.01	9.51±2.24	0.43
Mean daily chest drainage volume (mL)	191±75.5	128±80.9	0.17
Duration of indwelling catheter use (days)	6.25±1.71	5.88±2.04	0.56
Postoperative hospitalization duration (days)	7.31±2.21	6.52±3.37	0.39

### Postoperative pathological conditions

Among patients who underwent standard radical lung cancer resection, the complete resection ratio tended to be higher in the test group at 93.8% (15/16) than in the control group at 62.5% (5/8), although this difference did not reach statistical significance (*p*=0.053). Incomplete resections were typically due to extracapsular extension. Furthermore, two test group patients achieved clinical down-staging from stage IIIA to stage IIA for a down-staging ratio (DSR) of 12.5%, while none of the control group patients achieved down-staging (*p*>0.05); numbers of positive N2 lymph nodes and stations were comparable in the two groups (*p*>0.05) (Table 4).

**Table 4:** Postoperative pathological indicators

	Test group (n=16)	Control group (n=8)	*p* value
Lymph node metastases	3.69±6.37	5.63±5.21	0.47
Lymph node metastasis groups	1.63±1.03	2.25±1.98	0.31
Metastatic lymph node ratio	14.34±16.29%	20.53±17.90%	0.40
N2 lymph node metastases	2.31±3.79	3.37±3.11	0.50
N2 lymph node metastasis groups	1.06±0.85	1.26±1.48	0.68
Metastatic N2 lymph node ratio	13.45±14.29%	17.13±14.71%	0.56
Down-staging ratio	12.5%	0	0.30
Complete resection ratio	93.8%	62.5%	0.05

## DISCUSSION

NSCLC accounts for approximately 80% of all lung cancers, and 30%-35% of NSCLC cases are stage IIIA (N2). Neoadjuvant chemotherapy combined with other treatments is standard before surgery in patients with other stage IIIA cancers. Neoadjuvant chemotherapy typically reduces tumor infiltration into surrounding tissues and increases tumor shrinkage, thus rendering previously unresectable tumors resectable and often resulting in clinical down-staging, increased resection rates, and reduced resistance to chemotherapeutic agents [9]. However, some clinical trials have suggested that neoadjuvant chemotherapy has limited efficacy and fails to improve overall survival in lung cancer patients [10].

Endostar, a novel recombinant human endostatin developed in China, reduces tumor proliferation by inhibiting endothelial cell proliferation and migration, thus suppressing tumor vascularization and blocking the supply of nutrition and oxygen to tumor cells [11]. Endostar also normalizes tumor microcirculation by disrupting survival pathways in cancer cells, promotes the proliferation of perivascular hair cells and supporting cells, and enhances vascular nutrition supply, increasing the accessibility of tumor cells to chemotherapeutic agents and thus improving their efficacy [12–14]. Endostar and similar antiangiogenic therapies selectively attack active endothelial cells and are associated with only mild adverse effects in normal tissues. Reduced drug resistance in tumor cells enables Endostar to act synergistically with other molecular therapies and conventional chemotherapies to kill tumor cells through different antineoplastic mechanisms, which has led to an increase in the combination of antiangiogenic therapies with conventional chemotherapies [15].

Combining Endostar with NP neoadjuvant chemotherapy increased median TTP by 2.7 months, overall RR by 15.9%, and overall CBR by 9.3% compared to an NP regimen alone in advanced NSCLC patients [7]. Combined endostatin and chemotherapy treatments have also increased treatment efficacy for various advanced tumors [16–18]. For example, Endostar combined with neoadjuvant chemotherapy increased tumor response rates without increasing toxicity in breast cancer patients [19]. However, the safety, efficacy, and impact on surgical treatment of combining Endostar with neoadjuvant chemotherapy in NSCLC has not been investigated.

In this study, we evaluated combined treatment with Endostar and NP neoadjuvant chemotherapy in NSCLC for the first time. Combined treatment tended to increase response rates (RR) and clinical benefit rates (CBR) by 10% and 27.5%, respectively, compared to the NP monotherapy group; however, these differences did not reach statistical significance, likely due to the limited sample size. However, total response rates (TRR) increased significantly after combined treatment compared to chemotherapy alone (19.7% vs. 7.1%). Furthermore, two test group patients achieved clinical down-staging from stage IIIA to stage IIA; no control group patients achieved down-grading. Test group patients also had higher complete resection ratios, and slightly, albeit not statistically significantly, longer disease-free survival (DFS) and overall survival (OS), than control group patients. Together, these results indicate that combined anti-angiogenesis and neoadjuvant chemotherapy treatments are more effective than chemotherapy alone.

Several clinical studies have demonstrated that reduced cardiac function is a common adverse reaction to Endostar, and less-common adverse reactions include mild fever, rash, debilitation, dizziness, headache, chest distress, and diarrhea; combination of Endostar with chemotherapy did not increase the frequency or severity of adverse reactions [8, 20]. In addition, combining Endostar with NP results in synergistic effects without increasing adverse reactions to chemotherapy. Indeed, Endostar binds to highly-proliferative nucleolin, a vascular endothelial cell receptor, to effectively suppress tumor growth without increasing toxicity [21].

Studies indicate that vascular endostatin does not affect wound healing. Regarding hematological toxicity, clinical data indicate only a 0.61% incidence of grade I-II bleeding, and no occurrences of grade III-IV bleeding, after Endostar treatment [7]. Because there is no consensus regarding the optimal timing of Endostar treatment and its impact on surgery, we examined clinical indicators related to various aspects of surgery among patients who underwent standard radical resection of lung cancer within 4 weeks after combined Endostar and NP treatment. Compared to neoadjuvant chemotherapy alone, combined therapy had no effect on any surgery-related indicators, including intraoperative blood loss, mean postoperative daily chest drainage volume, and duration of postoperative indwelling catheter use.

In conclusion, we found that 2 cycles of Endostar combined with an NP regimen in stage IIIA-N2 NSCLC patients improved the short-term efficacy, as measured by TRR, of treatment compared to NP neoadjuvant therapy alone; other measures of short-term efficacy, including RR and CBR, and long-term efficacy, as measured by DFS and OS, also tended to increase after combined treatment. Furthermore, some patients who received combined treatment achieved clinical down-staging without an increase in adverse drug reactions, and there were no significant differences between the treatment groups in various perioperative clinical indicators. These results suggest that Endostar combined with NP neoadjuvant chemotherapy is a safe and effective therapeutic option for stage IIIA-N2 NSCLC patients.

## MATERIALS AND METHODS

### Patient characteristics

**Figure 3:**
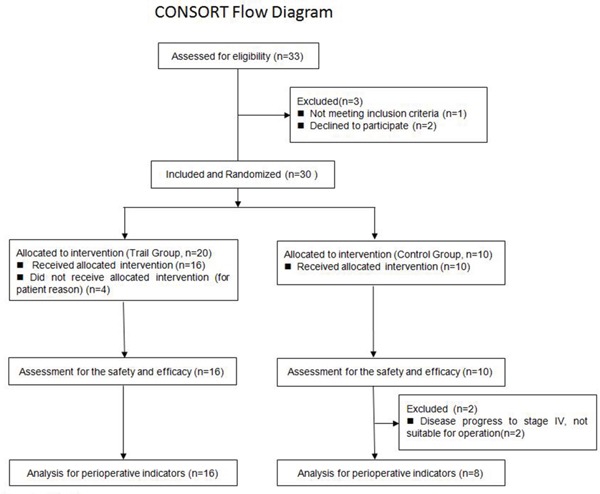
CONSORT Flow Diagram

This study was approved and supervised by the TianJin Medical University Cancer Institute and Hospital Ethics Committee. All patients signed informed consent forms, and the study complied with all ethical requirements (Figure 3). Inclusion criteria were as follows: (1) patients aged 18-70 years with histopathologically confirmed stage IIIA NSCLC (N2) upon auxiliary examination; (2) assessed as completely resectable upon imaging and laboratory examinations; (3) no previous chemotherapy or antiangiogenic therapy treatments; (4) presence of measurable target lesions; (5) absence of functional impairment of major organs; (6) generally normal hematology results, hepatic and renal function, and ECG. Exclusion criteria were as follows: (1) pregnant or lactating women, or women of child-bearing potential who were not using effective contraceptive measures; (2) severe infection; (3) severe cardiac disease; (4) uncontrolled neurologic or mental disorders; (5) severe diabetes; (6) evident tendency for excessive bleeding; (7) history of other tumors within the last 5 years.

A total of 30 patients with stage IIIA (N2) NSCLC selected according to the above criteria between April 2011 and December 2013 were randomly assigned to experimental groups; 20 were included in the test group and 10 in the control group. All patients were diagnosed with stage IIIA(N2) NSCLC by mediastinoscopic lymph node biopsies. Four patients dropped out of the study for personal reasons; treatment safety and efficacy were evaluated in the remaining 26 patients. General patient characteristics, including gender, age, and pathological type, were similar between the two groups (*p*>0.05) (Table 5).

**Table 5:** General characteristics of patients in whom efficacy was evaluated

Characteristic	Test group (n=16)	Control group (n=10)
Median age (range)	53 (45-68)	58 (36-63)
Gender		
Male	14 (87.5%)	9 (90%)
Female	2 (12.5%)	1 (10%)
Pathological type		
Adenocarcinoma	9 (56.2%)	6 (60%)
Squamous carcinoma	4 (25.0%)	3 (30%)
Other	3 (18.8%)	1 (10%)

### Treatment

In this randomized, controlled, open-label, explorative clinical study, patients were randomized at a 2:1 ratio into the test group, which received NP and Endostar, or the control group, which received NP chemotherapy alone. Endostar was administered at a dose of 7.5 mg/m^2^ (1.2 × 10^5^ U/m^2^) in 500 mL of normal saline via ivgtt at constant rate over 3-4 hours daily for 14 days; 25 mg/m^2^ vinorelbin was administered ivgtt on days 1 and 8, and 75mg/m^2^ cisplatin was administered iv on days 1-3 of each 21-day treatment cycle [7]. All test and control group subjects completed 2 treatment cycles. Efficacy was assessed 3 weeks after the completion of cycle 2, and standard surgical procedures occurred within 4 weeks after the completion of cycle 2.

### Evaluation variables

Response rate (RR), clinical benefit rate (CBR), and tumor regression rate (TRR) were the primary efficacy variables. Drug safety and toxicity and perioperative clinical indicators, including intraoperative blood loss, duration of indwelling catheter use, mean chest drainage volume, and lymph node dissection, were the safety variables. Disease-free survival (DFS) and overall survival (OS) were measured as secondary efficacy variables. Toxicity was assessed according to the WHO criteria, and efficacy was assessed based on RECIST 1.1 [22], where RR was defined as the percent of patients with complete response (CR) or partial response (PR) among all patients evaluated, CBR was defined as the percent of patients with CR, PR, or stable disease (SD), and TRR was defined as the proportion by which the longest dimension of the target lesion decreased. All patients underwent systemic physical examinations before they received treatment and after 2 cycles of drug therapy. All patients underwent enhanced chest CT scanning or PET-CT to evaluate treatment effects and abdominal and cervical ultrasound examinations, skull enhanced CT scanning of the skull, and ECT to exclude distant metastasis before and after the 2 treatment cycles.

### Follow-up

Routine chemotherapy and radiotherapy evaluations were conducted during the first follow-up visit. Patients follow-ups also occurred every 3 months for the first 2 years after treatment, and every 6 months thereafter.

OS was defined as the time from the first day of treatment to the date of death or the final follow-up. PFS was defined as the time from the first day of treatment to the date of local/regional recurrence, distant metastasis, or death.

### Statistical methods

All the statistical analyses were conducted using SPSS19.0 software. Normally distributed variables are presented as means ± standard deviation. Wilcoxon rank sum tests were used for comparisons of continuous variables. Chi-square tests were used for comparison of categorical variables. Log-Rank tests were used to compare of DFS and OS. All statistical tests were two-sided; *p*<0. 05 indicated statistically significant differences.
